# Recent Applications of Scanning Microscopy in Surface Engineering

**DOI:** 10.1155/2018/7546310

**Published:** 2018-05-14

**Authors:** Guosong Wu, Eun-Ha Choi, Paul K. Chu, Gheorghe Dinescu, Ranju Jung, Ying Zhao

**Affiliations:** ^1^Hohai University, Nanjing, China; ^2^Kwangwoon University, Seoul, Republic of Korea; ^3^City University of Hong Kong, Kowloon Tong, Hong Kong; ^4^National Institute for Laser, Plasma and Radiation Physics, Magurele, Ilfov, Romania; ^5^Shenzhen Institutes of Advanced Technology, Chinese Academy of Sciences, Shenzhen, China

Surface engineering is a multidisciplinary field that combines physics, chemistry, materials science, biology, and mechanical engineering. Metals, ceramics, and polymers can be modified to have novel properties by surface modification techniques such as electrochemical deposition, chemical conversion, and plasma treatment. Scanning microscopy is widely used to explore the microscopic world, and comprehensive information can be acquired by scanning microscopy to reveal the fundamental aspects of physics, chemistry, and biology on surfaces. The utilization of scanning microscopy in surface engineering provides many opportunities to improve surface modification processes and understand the associated mechanisms.

The aim of this special issue is to establish a platform for physicists, chemists, biologists, materials scientists, and engineers to share and disseminate recent applications of scanning microscopy in surface engineering. This special issue contains 17 research papers and one review paper representing the latest research in surface engineering.

Scanning electron microscopy (SEM) has already become a general analytical tool to observe the surface morphology of various materials and is currently playing a very important role in surface engineering. In this special issue, there are 13 papers about the application of SEM and the content is quite extensive, involving common structural materials, emerging biomaterials, and promising energy materials. For example, magnesium alloys are recommended for bone repair due to their natural degradation in the human body and mechanical properties similar to those of bones. It has thus become a hot research topic in the field of biomaterials. Usually, the development of biomedical Mg-based materials is closely related to surface treatment because their degradation rate has to be carefully controlled. In this special issue, C. Liu et al. present a review to introduce recent progress and Y. Su et al. introduce their research about the *in vitro* degradation behavior of Mg-Ca-based alloys with and without coatings.

Besides scanning electron microscopy, this special issue contains 5 papers about the recent application of other scanning microscopic techniques. L. Zhang et al. used atomic force microscopy (AFM) to investigate Langmuir monolayers, and X. Fu et al. applied AFM to study the nanoscale characteristics of the nanoindented ferromagnetic shape memory thin film. H. Zhang et al. presented an image postprocessing framework for scanning tunneling microscopy (STM) to reduce strong spurious oscillations and scan line noise at fast scanning rates with preserving the features. Y. Fan et al. used a computed tomography (CT) scanner to observe human organs and provided a method to find the same imaging plane in images obtained during separate scanning sessions. This novel approach may offer clinicians knowledge about the morphology and structure of a lesion through cross-sectional images reconstructed along arbitrary axes. This potentially reduces the misdiagnosis rate when cross-sectional images are interpreted.

In summary, the contributed papers cover several general aspects of surface engineering and technologies. We try our best to present latest applications of scanning microscopy in this field and hope that our endeavor will provide the authors with inspirations for their future research.

